# A Guide to Single-Cell Transcriptomics in Adult Rodent Brain: The Medium Spiny Neuron Transcriptome Revisited

**DOI:** 10.3389/fncel.2018.00159

**Published:** 2018-06-15

**Authors:** Hanson Ho, Matt De Both, Ashley Siniard, Sasha Sharma, James H. Notwell, Michelle Wallace, Dino P. Leone, Amy Nguyen, Eric Zhao, Hannah Lee, Daniel Zwilling, Kimberly R. Thompson, Steven P. Braithwaite, Matthew Huentelman, Thomas Portmann

**Affiliations:** ^1^Circuit Therapeutics, Inc., Menlo Park, CA, United States; ^2^Neurogenomics Division, Translational Genomics Research Institute, Phoenix, AZ, United States

**Keywords:** single-cell RNA sequencing, fluorescence-activated cell sorting, striatal medium spiny neurons, basal ganglia, dopamine receptors, Cholinergic Receptor Muscarinic 4

## Abstract

Recent advances in single-cell technologies are paving the way to a comprehensive understanding of the cellular complexity in the brain. Protocols for single-cell transcriptomics combine a variety of sophisticated methods for the purpose of isolating the heavily interconnected and heterogeneous neuronal cell types in a relatively intact and healthy state. The emphasis of single-cell transcriptome studies has thus far been on comparing library generation and sequencing techniques that enable measurement of the minute amounts of starting material from a single cell. However, in order for data to be comparable, standardized cell isolation techniques are essential. Here, we analyzed and simplified methods for the different steps critically involved in single-cell isolation from brain. These include enzymatic digestion, tissue trituration, improved methods for efficient fluorescence-activated cell sorting in samples containing high degree of debris from the neuropil, and finally, highly region-specific cellular labeling compatible with use of stereotaxic coordinates. The methods are exemplified using medium spiny neurons (MSN) from dorsomedial striatum, a cell type that is clinically relevant for disorders of the basal ganglia, including psychiatric and neurodegenerative diseases. We present single-cell RNA sequencing (scRNA-Seq) data from D1 and D2 dopamine receptor expressing MSN subtypes. We illustrate the need for single-cell resolution by comparing to available population-based gene expression data of striatal MSN subtypes. Our findings contribute toward standardizing important steps of single-cell isolation from adult brain tissue to increase comparability of data. Furthermore, our data redefine the transcriptome of MSNs at unprecedented resolution by confirming established marker genes, resolving inconsistencies from previous gene expression studies, and identifying novel subtype-specific marker genes in this important cell type.

## Introduction

The structural complexity of the human brain presents challenges to a mechanistic understanding of its development and function. Single-cell transcriptomics hold great promise in resolving heterogeneous tissues at cellular resolution and providing insights into cell type-specific aspects of brain function in healthy and disease states. The technology reached a mature level with integration of RNA sequencing technology and application to the study of neuronal and non-neuronal tissues is taking off rapidly including initiatives toward building a cell atlas of the body (Ecker et al., 2017; Rozenblatt-Rosen et al., 2017). In the adult central nervous system (CNS) however, application of single-cell RNA sequencing (scRNA-Seq) has largely focused on regions with well-characterized neuronal circuit architecture, including the cerebral cortex (Zeisel et al., 2015; Tasic et al., 2016) (for review see Molyneaux et al., 2007). About neuronal cell types beyond the cerebral cortex much less is known. Single-cell data available from CNS regions other than cortex include striatal medium spiny neurons (MSN). However, with the exception of a recent report by Gokce et al. (2016) using scRNA-Seq across striatal cell types (Gokce et al., 2016), these studies provide limited transcriptome coverage due to their reliance on multiplexed qPCR technology (Portmann et al., 2014; Fuccillo et al., 2015). This level of understanding is imperative as many subcortical neuronal subtypes including striatal MSN support critical information processing and have been implicated in various neurological disorders. Striatal MSN expressing either D1 or D2 dopamine receptors form the entry-point to the two functionally antagonistic neuronal pathways of the basal ganglia (Kravitz et al., 2012) (for review see Kreitzer and Malenka, 2008). D1 MSN project along the direct basal ganglia pathway to the internal globus pallidus and substantia nigra pars reticulata, major output structures of information processing through the basal ganglia nuclei. The direct pathway has been referred to as providing positive signals of re-enforcement that increase the likelihood for initiation of behaviors. D2 MSN project along the indirect basal ganglia pathway through the external globus pallidus and subthalamic nucleus. The indirect pathway is thought to provide an inhibitory signal referred to as avoidance or punishment that reduces the likelihood of action initiation. Both MSN subtypes are strongly modulated by dopamine, the striatal release of which shifts the balance between the direct and indirect pathway through activation of G_αs_-coupled D1 receptors and G_αi_-coupled D2 receptors. In this context, MSN function and imbalance is highly relevant to disorders of the basal ganglia and the dopamine system including Huntington’s disease, Parkinson’s disease, drug addiction, schizophrenia, ADHD and other psychiatric and neurodegenerative conditions.

Based on their critical importance it is of great interest to understand the molecular composition of the different MSN subtypes in order to gain mechanistic insights to function and derive drug targets to treat dysfunction. Thus, MSN have been the focus of previous studies attempting to resolve their subtype-specific transcriptome (Heiman et al., 2008). Until recently, BacTRAP technology has provided the most comprehensive differential gene expression profiles between D1 and D2 MSN (Doyle et al., 2008). The technology made use of an EGFP-tagged ribosomal subunit (L10) to isolate cell type-specific pools of translated mRNAs from whole tissue extracts. Despite a high degree of overlap with known MSN subtype-specific marker genes and valuable extension of transcriptome understanding, BacTRAP data sets displayed differentially expressed genes between D1 and D2 MSN that are not easily reconciled with other available gene expression data from striatum, including ISH data from the Allen Brain Atlas (Lein et al., 2007). For example, the transcription factor Lhx8 was reported as D2 MSN-specific although other studies suggested that this gene is pivotal for development of cholinergic interneurons in striatum and not co-expressed with Darpp32, a marker specifically expressed by MSN (Zhao et al., 2003; Mori et al., 2004; Fragkouli et al., 2009). Also, reported D1 MSN-specific expression of the Cholinergic Receptor, Muscarinic 4 (*Chrm4*) was not confirmed by BACTRAP technology (Bernard et al., 1992; Yan et al., 2001), while genes previously thought to be exclusive to glutamatergic projection neurons, including the known cortical excitatory marker *Tbr1*, were reported to be specific to D1 MSN (Schuurmans et al., 2004; Guillemot et al., 2006; Hevner et al., 2006). These inconsistencies raised the possibility of false negative and false positive differentially expressed genes between D1 and D2 MSN subtypes as a result of technologies that lack single-cell resolution.

A major reason for the limited availability of single-cell data from the adult brain is the technical challenge of isolating single cells in the first place, associated with the heavy interconnectivity of neurons that form both local networks and distant projections within and beyond the brain. Specific factors that contribute to this challenge in single cell isolation include the severe disruption of cellular connectivity, the time frame needed to proceed from isolation of the brain to the generation of cDNA from the minute amounts of RNA in a single cell (typically 1–10 pg), and the reproducibility of specific steps in current single-cell isolation procedures. Several groups have presented protocols for extraction of intact cells from adult rodent brain (Rusznák et al., 2001; Kuehl-Kovarik et al., 2003; Bergmann et al., 2005; Okada et al., 2011; Legroux et al., 2015). Some protocols aimed at isolation of major CNS cell types including hippocampal pyramidal neurons and striatal MSN for the purpose of primary neuronal cell culture have a strong focus on cell viability. Therefore, they may present an ideal starting point for single-cell transcriptomics (Brewer and Torricelli, 2007; Ena et al., 2013). Many protocols include common steps to obtain single cells from adult rodent brain as a starting point. First, acute brain slices are generated using a vibrating microtome (vibratome) followed by manual dissection of the brain regions of interest. Second, enzymatic digestion loosens the extracellular matrix and prepares the cells for the next step of mechanical trituration. Third, mechanical trituration using repeated pipetting of pieces of tissue results in a single-cell suspension ready for collection of single cells by fluorescence-activated cell sorting (FACS) or other methods. Particularly, the third step involving mechanical trituration by Pasteur pipettes fire-polished to different inner diameters is a major impediment to standardization across labs due to highly subjective parameters of manual fire-polishing, and trituration force/intensity applied.

A further challenge to single-cell isolation from adult brain is the high fraction of debris particles from fragmented myelin and neuropil. This is in contrast to cell cultures or embryonic brain from which the majority of isolated particles are relatively intact cells. The fact that a minor fraction of the adult brain’s cellular material constitutes actual cell bodies after dissociation highly reduces the efficacy of single-cell isolation by FACS. Some groups have tried to work around this problem by adding an additional step to their isolation protocol involving a density gradient centrifugation with the aim of enriching for cell bodies while reducing debris (Brewer and Torricelli, 2007; Ena et al., 2013). However, a systematic assessment of the efficacy and usefulness of each of the many steps involved in single cell isolation is, to our knowledge, lacking.

Finally, some brain structures are not easily identified due to the absence of visible anatomical landmarks. This can render precise and reproducible isolation of specific nuclei for single-cell analysis impossible or requires additional steps of tissue-fixation for laser-dissection/capture. Ideally, a cell labeling method in anatomically less well-defined brain structures would allow use of established methods of stereotaxic surgery including commonly used coordinate systems from standard brain atlases.

In summary, all these factors have an impact on efficacy and reproducibility of single-cell isolation from specific regions of the adult brain tissue. As a consequence, they affect comparability of currently established scRNA-Seq data sets in the field, and this despite increased efforts for standardization and improved QC steps in the actual scRNA-Seq protocols. Therefore, development of efficient, reproducible methods for isolation, enrichment and unambiguous identification of cell somas from brain tissue are highly needed.

Here we designed a protocol for efficient and reproducible single cell isolation from adult rodent brain and determine the factors that define these criteria. We developed solutions to increase reproducibility of critical steps including mechanical trituration and cell sorting by FACS. We further present a method for cell labeling in precise anatomical sub-regions by using nuclear stains compatible with stereotaxic injection and standard coordinate systems used in the neuroscience field. We then applied our optimized protocol to the study of D1 and D2 MSN, specifically from dorsomedial striatum and sequenced their transcriptome by scRNA-Seq. Comparison with previous D1 and D2-specific gene expression data sets removes previously reported genes that were likely false positives and importantly, reveals several additional MSN subtype-specific genes that might be of interest for further understanding the function of this important cell type.

## Methods

### Animal Studies

All experiments were carried out in accordance with Guidelines for Animal Care and Use at Circuit Therapeutics. Animal research at Circuit Therapeutics is conducted in accordance with the National Research Council’s Guide for the Care and Use of Laboratory Animals, and the Office of Laboratory Animal Welfare’s Public Health Service Policy on the Humane Care and Use of Laboratory Animals. All research involving research animals is reviewed and approved by the Institutional Animal Care and Use Committee (IACUC) at Circuit Therapeutics Inc., United States.

Animal strains used include C57Bl6/J, and BAC-transgenic mouse lines Drd1a-TdTomato [The Jackson Laboratory, B6.Cg-Tg(Drd1a-tdTomato)6Calak/J] and Drd2-EGFP [MMRRC, Tg(Drd2-EGFP)S118Gsat/Mmnc] (Gong et al., 2003; Ade et al., 2011).

### Tissue and Single Cell Collection

Isoflurane-anesthetized animals at age 8–12 weeks were perfused with ice-cold artificial cerebrospinal fluid (ACSF). Brains were immediately dissected and mounted on a PELCO easiSlicer vibrating microtome (Ted Pella) in HABG medium containing Hibernate A (Brainbits LLC, #HA), B27 supplement (Life Technologies, #17504), and 0.5mM Glutamax (Thermo Fisher Scientific, #35050-061) (Brewer and Torricelli, 2007). All tissues were sectioned coronally. Regions of interest were dissected under a Leica M165 FC Stereomicroscope with Fluorescence (Leica), transferred to a 15 ml falcon tube with pre-warmed papain solution and immediately digested for 30 min at 30°C in 2 mg/ml papain dissolved in Hybernate A Minus Calcium medium (Brainbits, #HA-Ca), 0.125x Glutamax while gently shaking. Importantly, the papain solution also contained the cell permeable, nuclear dye to label cell bodies, either DRAQ5 (5 mM solution at 3000-fold dilution, Thermo Fisher, #PI62251) or Hoechst 33258 (10 mg/ml at 1000-fold dilution, Thermo Fisher, #H3569). Papain solution was replaced by 2 ml HABG medium and tissue pieces mechanically triturated 10 times using stainless steel needles with decreasing inner diameter (McMater-Carr, #75165A754, #75165A757, #75165A755, #75165A761). In between trituration steps, remaining tissue pieces were allowed to settle for 2 min and the top 1 ml of solution transferred through a 45 μm cell strainer to a new tube and replaced with fresh HABG medium for following trituration with the next smaller needle size.

Optional step: Collected single-cell fractions from different trituration steps were applied to density gradient adapted from Brewer and colleagues (Brewer and Torricelli, 2007) and spun at 1900 rpm (786 g) in a cooled Sorvall Legend XTR table-top centrifuge with a TX-750 bucket rotor (Thermo Fisher, #75003607) for 30 min at 4°C, applying slow acceleration/deceleration. The top layer containing HABG medium with low density particles including debris was removed.

Cells were resuspended in a total of 14 ml HABG containing a suitable dead stain, propidium-iodide (at 1000-fold dilution, Sigma-Aldrich, #P4864) if the cell-permeable label was Hoechst 33258, or DAPI (10mg/ml stock solution at 1000-fold dilution, Santa Cruz Biotechnology, #sc-3598) if the cell-permeable label was DRAQ5. Cells were precipitated by centrifugation at 1100 rpm (264 *g*) at 4°C, carefully resuspended in HABG and filtered using a 45 μm cell strainer before FACS.

For the comparison of pronase and papain treatments, the tissues for the pronase group were treated with 1 mg/ml pronase (Sigma, Cat#P6911-1G) in carbogen-bubbled ACSF for 70 min at room temperature, and as previously described (Tasic et al., 2016). DRAQ5 staining was done during the pronase digestion, comparable to the protocol using papain.

Cell sorting was performed on BD FACSAria II, BD Influx or Sony SH800 instruments. Combinations of cell-permeable (to label cell bodies) and cell-impermeable dyes (to label disrupted cells) were used according to availability of filters on each instrument and as indicated in the corresponding figures. Cells were collected into cooled 96-well PCR plates (Bio-Rad, #HSP-9601) containing 2 μl nucleic acid-free water, 2% Triton-X100, and RNAse inhibitor (Thermo Fisher Scientific, #PR-N2611). MSN included in the scRNA-Seq study originated from three independent experiments and a total of three animals.

### Stereotaxic Surgery

Stereotaxic injections were performed using a 10 μL Hamilton syringe and a 32G needle attached to a WPI Ultra Micro Pump. 8- to 12-week-old, double-transgenic Drd1a-TdTomato and Drd2-EGFP female mice were injected 150–300 nL of a mixture of India ink (20-fold dilution, Thermo Fisher, #NC9903975) and Hoechst 33258 (10 mg/ml at 100-fold dilution) diluted in physiological saline solution. Bilateral injection in the dorsomedial striatum was done using the following coordinates from bregma at skull level: M/L ± 1.5, A/P + 0.8, D/V - 3.5. Injections were followed by a 5 min waiting period to let the marker solution penetrate the tissue. After 5 min the syringe was lifted approx. 500–750 μm followed by a second 5 min period before carefully pulling out the needle completely. After injection, animals were allowed to incubate for 30 min under anesthesia before perfusion with ice-cold ACSF and collection of the brain.

### NGS Library Generation and Single-Cell RNA-Seq

The SMARTer Ultra Low Input RNA Kit for Sequencing – v3 (Clontech Laboratories, #634851) was used to generate cDNA from a single cell following the manufacturers protocol. The cDNA was then visualized and quantified using the High Sensitivity DNA assay on a 2100 Bioanalyzer (Agilent Technologies, #5067-4626). Sequencing libraries were prepared with 2ng of cDNA using the Low Input Library Prep Kit (Clontech Laboratories, #634947) following the manufacturers protocol. Final libraries were validated via the High Sensitivity DNA assay on a 2100 Bioanalyzer (Agilent Technologies, 5067-4626) and quantified by qPCR using Kapa’s Library Quantification Kit (Kapa Biosystems, #KK4824) on the 7900HT (Applied Biosystems). Libraries were pooled prior to sequencing by 75 bp paired-end reads using reagents from the MiSeq 150 cycle kit v3 (Illumina, #MS-102-3001).

### RNA-Seq Analysis

Illumina BCL files were converted and demultiplexed (bcl2fastq 2.18) using default parameters.

A custom mouse reference genome was created by adding EGFP and TdTomato sequences to the mouse reference genome mm10/GRCm38 and gene annotation version GRCm38.75. *Gpr52* was added to confirm previous findings.

Fastq files were aligned to the custom mouse reference genome (STAR 2.5.0). Sequencing and RNA quality control reports were generated (FastQC 0.11.5 and Qualimap 2.2.1). Aligned reads were summarized as gene-level counts (featureCounts 1.5.1). Pairwise differential expression was conducted between groups with the R package DESeq2 (v1.14.1).

For analysis of the data by Gokce et al. (2016) Fastq files were aligned to the mm10/GRCm38 mouse reference genome using STAR 2.5.0. Aligned reads were summarized as gene-level counts (featureCounts 1.5.1) independently for both RefSeq and Ensembl gene set annotations. Furthermore, aligned reads from cells unambiguously identified as D1 or D2 MSNs based on marker gene expression were pooled and converted into wiggle tracks using the UCSC kentUtils.

### Data Availability

All scRNA-Seq data in this study is available at Gene Expression Omnibus under the accession code GSE112177.

## Results

### Acute Single Cell Isolation From Brain

**FIGURE 1:**
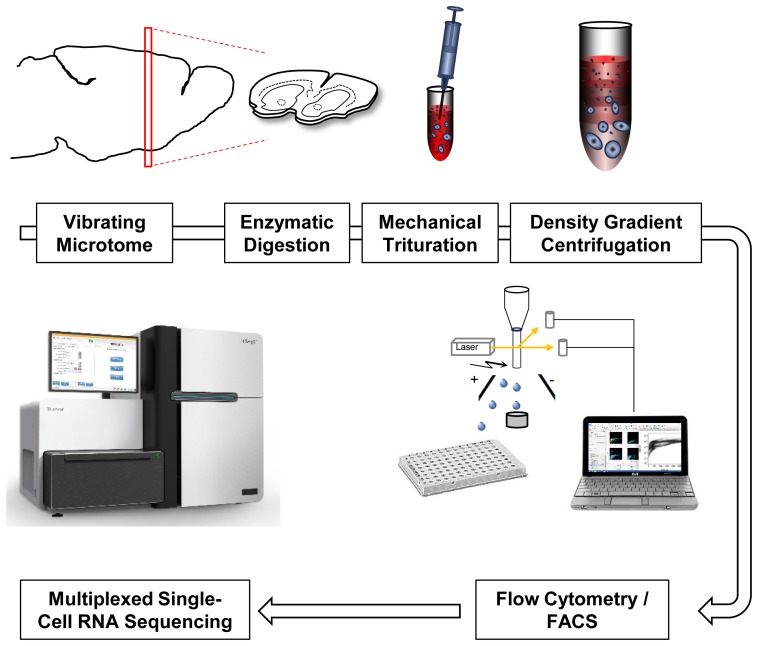
Single-cell RNA-Seq work flow. Vibratome sections are enzymatically digested and mechanically triturated resulting in a single cell suspension. Density gradient centrifugation removes cellular debris. Single cells are collected by fluorescence-activated cell sorting (FACS). After library generation and quality control, single-cell transcriptomes are sequenced using Illumina protocols.

As a starting point for developing a reproducible and efficient single-cell isolation protocol from adult rodent brain we took two previous studies that aimed at extraction of major, postmitotic CNS cell types including hippocampal pyramidal neurons and striatal MSN (Brewer and Torricelli, 2007; Ena et al., 2013). **Figure 1** provides a scheme of critical steps involved in these protocols (**Figure 1**). We perfused 8- to 12-week-old wild-type mice (C57BL6/J) with ice-cold ACSF, and immediately mounted them on a vibratome for preparation of acute slices. A particularly useful resource about factors affecting health of acute brain slices is provided by http://www.brainslicemethods.com. Brain slices were then subjected to enzymatic digestion. A commonly used enzyme for digestion of adult and juvenile neural tissue is papain (Brewer and Torricelli, 2007). However, other enzymes including Proteinase type XIII and pronase have been used in similar protocols (Ena et al., 2013; Tasic et al., 2016). A comparison of tissue exposure to papain (30 min, 2 mg/ml, 30°C) and Proteinase type XIII (20 min, 1.5 mg/ml, 30°C) similar to previous reports suggested better performance of papain based on smooth cell morphology and increased viability (Supplementary Figure S1).

### Assessment of Cell Viability

**FIGURE 2:**
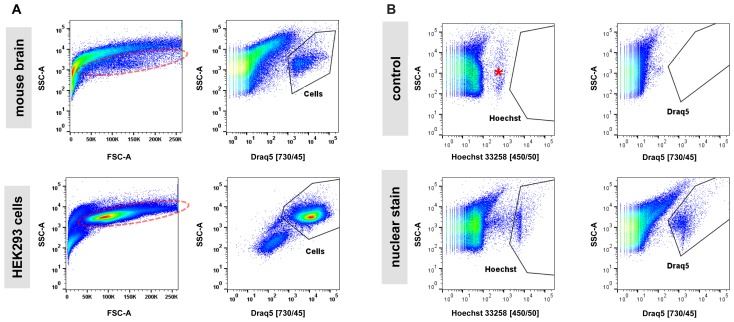
Nuclear dyes facilitate isolation of adult brain cells by FACS. **(A)** Top: Single-cell suspensions from adult brain tissue display a broad, indistinctive smear in the standard FSC/SSC plot used to identify cells by FACS due to (1) the overwhelming presence of debris from neuropil fragments and (2) the morphological diversity of CNS cell types (left). Introduction of the nuclear stain DRAQ5 allows for efficient isolation of cell bodies based on the presence of a cell nucleus (right). Bottom: Single-cell suspensions from cultured HEK293 cells allow isolation of single cells due to higher morphological homogeneity and the absence of debris present in dissociated brain tissue (left). Furthermore, a nuclear stain can enhance isolation by FACS for cultured cells (right). Photomultiplier tube (PMT) voltage for cell culture samples were set at 50% compared to adult brain cells due to the larger size of the cultured HEK293 cells. **(B)** Comparison of two common nuclear stains, Hoechst 33258 (left) and DRAQ5 (right), for single-cell isolation of adult CNS cells. Increased background fluorescence of a group of particles in the UV spectrum (asterisk) results in a lower signal/noise ratio for the nuclear dye Hoechst 33258 compared to DRAQ5.

Next, we examined the impact of various steps in the single-cell isolation procedure on cell viability based on propidium-iodide (PI) incorporation and quantification by flow cytometry. In cell suspensions derived from blood or cell culture, the majority of obtained particles measured by flow cytometry are cell bodies. The assessment of the live/dead cell ratio is therefore directly calculated as the ratio of PI-positive (dead) particles versus all other (live) particles. In samples from adult mouse brain analyzed by flow cytometry, we noticed that (1) the majority of particles are in a size range smaller than what is usually expected from cultured cells, and (2) that instead of forming a narrow-range, distinct cloud in a typical scatter plot of forward (FSC) versus side scatter (SSC), what we perceived as cells appeared to spread across a wide size range (**Figure 2A**). This is in contrast to single-cell suspensions from cell culture that typically show a distinct cloud of cells in FSC versus SSC plots representing greater than 80% of detected particles. These observations were not unexpected given the heterogeneous cellular composition of the adult mouse brain. However, measuring a precise live/dead cell ratio required unambiguous identification of cell bodies over debris. Since every relatively intact cell body from brain is expected to contain a nucleus, we tested whether incorporation of a nuclear stain in our protocol allowed for distinction of cell bodies from debris. Most available nuclear stains are not membrane permeable (e.g., propidium-iodide and 4,6-Diamidino-2-phenylindole, DAPI) and are thus unsuitable for staining live cells. Exceptions include Hoechst 33258 (bis-Benzimide) and DRAQ5 nuclear stains (Smith et al., 1999; Lanuti et al., 2012). We determined that including either Hoechst 33258 or DRAQ5 in the 30-min enzymatic digestion of brain slices allowed for unambiguous distinction of nucleated cell bodies from debris. Application of a nuclear stain to single-cell suspensions from cultured HEK293 cells showed a superior separation of cells from debris suggesting that even in relatively homogenous samples from cell culture the isolation of cells based on nuclear stain may outperform classic FSC/SSC-based gating (**Figure 2A**). Consistent with decreasing light absorption in live tissue from UV to the far-red spectrum, DRAQ5 (maximum excitation at 646 nm) was able to separate cell bodies more efficiently from higher-range auto-fluorescent debris than Hoechst 33258 (maximum excitation at 352 nm, **Figure 2B**). In summary, our findings confirmed the considerable heterogeneity of adult brain cells in suspension across a wide size spectrum. They further suggest that the commonly applied gating for cells in an FSC/SSC plot is not practicable for sorting of cells isolated from adult mouse brain, as cell bodies and debris overlap to a considerable degree in this type of analysis.

### Parameters Affecting Cell Isolation Efficacy and Cell Viability

**FIGURE 3:**
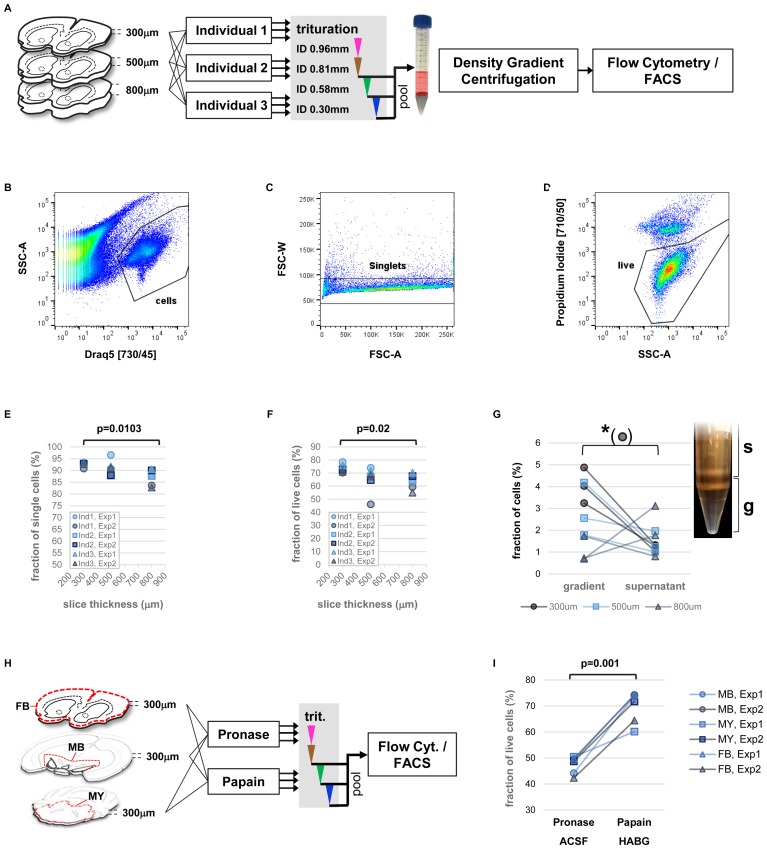
Precise measurements of critical parameters for single-cell isolation. **(A)** Schematic view of experimental design. Key variables included diameter of tissue slices and performing individual. Two independent experiments were performed. **(B–D)** Examples of flow cytometry data: identification of cell bodies (**B**, based on nuclear dye DRAQ5), singlets (**C**, within cells), and live single cells (**D**, based on propidium-iodide, within singlets). **(E,F)** Impact of tissue slice thickness, performing individual (Ind), and experiment (Exp) on yield of singlets **(E)**, and live single cells **(F)**. Nested ANOVA was performed with individuals and experiments treated as replicate groups. **(G)** Effect of density gradient centrifugation on cell enrichment versus debris. Paired *t*-test was performed. Inset shows the density gradient (g) and supernatant (s) containing cellular particles (white bands). **(H)** Schematic view of experimental design for comparison of enzyme treatment. Slices of forebrain (FB), ventral midbrain (MB), and parts of hindbrain (myencephalon, MY), were treated with papain (in HABG buffer) or pronase (in carbogen-bubbled ACSF). **(I)** Flow cytometry measurement of live single cells based on DAPI incorporation comparing treatment with papain and pronase. Two-way ANOVA.

The addition of a nuclear stain for efficient identification and separation of cell bodies from debris enabled us to precisely assess the fraction of single cells as well as the live/dead cell ratio obtained from single cell isolation of adult mouse brain. We therefore set out for a multi-parameter analysis of our protocol including vibratome sectioning, mechanical trituration, as well as the human factor potentially playing a role in the variability of cell viability (**Figure 3A**). In order to further improve on reproducibility and because initial experiments did not indicate any consistent difference we replaced manually fire-polished Pasteur pipets with commercially available, blunt steel needles mounted on 3 ml Luer-lock syringes (not shown). Three individuals were each assigned three forebrain hemispheres of different thickness (300, 500, 800 μm) after papain digestion. All individuals applied an identical trituration protocol adapted from Brewer and Torricelli consisting of 10-times repeated trituration each with needles of decreasing inner diameter (ID 0.96, 0.81, 0.58, 0.3 mm) in a total volume of 2 ml HABG medium that replaced the papain after enzymatic digestion of the brain tissue (Brewer and Torricelli, 2007). The only additional instruction provided to the participants was to avoid foam generation due to under-pressure in the syringe, and to keep the needle tip immersed in the cell suspension at all times to avoid introducing air bubbles. After trituration with the ID 0.81 mm and ID 0.58 mm needles remaining pieces of tissue were allowed to settle for at least 2 min. Subsequently, 1 ml of surface HABG with single cells was collected and filtered through a 45 um cell strainer. To recover the total volume of 2 ml for the following trituration step, 1ml of fresh HABG was added.

Analysis of fractions of single cells (singlets), and live cells across parameters (slice thickness, performing individuals, experiment) by flow cytometry revealed a predominant effect of brain slice thickness on protocol performance (**Figures 3B–D**). Specifically, significant differences in the fraction of singlets and live cells were found, with highest yield for thinner brain slices (**Figures 3E,F**, nested ANOVA *p* = 0.0103 and *p* = 0.02, respectively). Despite the manual trituration steps involved, the human factor did not play a significant role. In order to assess the effect of each trituration step on the protocol performance, we repeated the experiments for comparing isolated fractions of cell suspension after each step of trituration with decreasing inner needle diameter (Supplementary Figure S2A, ID 0.81, 0.58, and 0.3 mm). The results confirmed again slice thickness the critical parameter for the fraction singlets and live cells (Supplementary Figures S2B,C, ANOVA *p* = 0.0095 and *p* = 0.03, respectively). Our findings therefore identified vibratome sectioning and brain slice thickness a critical step for optimization of cell viability during single-cell isolation from adult rodent brain.

Next, we investigated the utility of a density gradient centrifugation for removal of debris and enrichment of cell bodies. Although this step adds in the range of 30 additional minutes to the protocol (Brewer and Torricelli, 2007; Ena et al., 2013), it might provide a benefit particularly for isolation of rare cell types as it increases speed and accuracy of automated cell sorting. An enrichment of cell bodies over debris results in increased efficacy and decreased electronic abort rate due to conflicting events when collecting cells by FACS. In order to test the usefulness of a density gradient centrifugation we adapted a simplified version of a density gradient previously developed for enrichment of hippocampal pyramidal neurons (Brewer and Torricelli, 2007). The gradient consisted of two layers with distinct water/iodixanol volume ratios. This approach allowed visible separation of components from the triturated cell suspension (**Figure 3G**). Microscopic inspection further suggested that the high-density bottom layer contained an increased number of large particles compared to the material accumulating above the top layer of the gradient, which mostly contained smaller particles (Supplementary Figure S2D). Nevertheless, we visually identified smaller cells in this upper fraction. Subsequent quantification of the cell/debris ratio in both fractions by flow cytometry showed a significantly increased cells/debris ratio in the gradient layer compared to supernatant (ANOVA *p* = 0.013). However, the effect was most pronounced at thinner brain slice thickness (ANOVA interaction *p* = 0.0077). The results support our observation by microscopy that smaller cell types might be lost with debris removal by density gradient centrifugation. The findings further show that the brain slice thickness has also an impact on the efficacy of debris removal by density gradient centrifugation, possibly due to different outcome of particle size depending on starting thickness of tissue material. In general, we recommend to carefully optimize or omit density gradient centrifugation if the protocol is applied to novel cell types of unknown density or to particularly small cells.

Finally, we compared our optimized protocol to a more recent and popular protocol, which uses the enzyme pronase to support tissue dissociation (Hempel et al., 2007; Tasic et al., 2016). In this protocol, tissue slices are treated with 1 mg/ml pronase in carbogen-bubbled ACSF at room temperature for 70 min. Since it is unclear whether efficacy of different enzyme treatments might depend on tissue composition, such as ratio of gray and white matter, we compared three brain regions across the brain in this experiment, forebrain, ventral midbrain, and parts of the myencephalon (**Figure 3H**). Flow cytometry results showed an increased yield of intact cells based on incorporation of the membrane-impermeable dye DAPI for treatment with papain versus pronase (**Figure 3I** and Supplementary Figure S2E, two-way ANOVA *p*_enzyme_ = 0.001) with no significant effect across brain regions (*p*_region_ = 0.7726). This finding suggests superior performance of papain for gentle dissociation of tissue from adult rodent brain.

### Isolation of D1 and D2 MSN From Dorsomedial Striatum Based on Stereotaxic Coordinates

**FIGURE 4:**
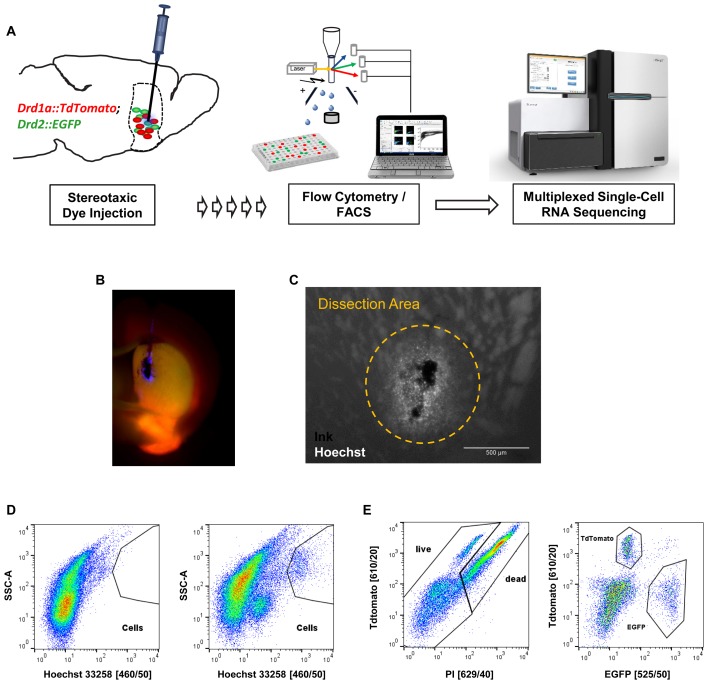
*In vivo* application of the cell isolation protocol for region-specific scRNA-Seq based on stereotaxic coordinates. **(A)** Schematic workflow for labeling cells *in vivo* in a cell type- and region-specific manner. BAC-transgenic reporters label the two major striatal MSN subpopulations based on their expression of the D1 and D2 dopamine receptors. Stereotaxic injection of the nuclear dye Hoechst 33258 allows specific labeling of cells in the DMS. Cells are isolated by FACS and RNA-Seq libraries produced for downstream Illumina sequencing. **(B)** Fluorescence microscopy image of a coronal section showing the right hemisphere of a double transgenic animal injected with a mixture of India ink (for visual guidance of tissue dissection) and Hoechst 33258 in the dorsomedial striatum. **(C)** Magnified view of an injection site. Note that the small molecule Hoechst 33258 spreads slightly further than the larger carbon particles contained in India ink. The approximate dissection area is indicated. **(D)** FACS data (comparable particle numbers) showing nuclear stain by Hoechst 33258 from a non-injected C57BL/6 control animal (left) and an injected double-transgenic animal (right). **(E)** Left: cells based on Hoechst 33258 gating shown in **(D)** assayed for cell death based on propidium-iodide (PI). Due to partial overlap of TdTomato and propidium-iodide with available filters, the gating was done setting TdTomato against propidium-iodide channels. Right: live cells based on left panel show Drd1a-TdTomato and Drd2-EGFP labeled MSN subpopulations within the dorsomedial striatum.

Next, we applied our optimized protocol to the isolation and characterization of striatal MSN. In order to specifically label MSN subtypes we used mice that express both Drd1a-TdTomato and Drd2-EGFP fluorescent reporter transgenes that label direct and indirect pathway projecting MSN, respectively (**Figure 4A**). Initial cell collection by FACS using DRAQ5 to identify cells, and EGFP and TdTomato to identify MSN subtypes, yielded high numbers of MSN and cell viability comparable to previous experiments (Supplementary Figure S3). Given that the striatum is a relatively large structure with several functional subdivisions we next tested whether we could use our nuclear labeling strategy to isolate MSN from a specific striatal subregion (Everitt and Robbins, 2005; Everitt and Heberlein, 2013). MSN in the dorsomedial part of the striatum (DMS) have been implicated in motor control through the basal ganglia circuits, in contrast to reward-related and addictive behaviors (controlled by ventral striatum) and thus may be of particular importance for motor symptoms including those found in Parkinson’s disease (Kravitz et al., 2010). In order to obtain reproducible intra-striatal region-specificity to the DMS, double-transgenic animals were injected with a mixture of India Ink and Hoechst 33258 using previously established stereotaxic co-ordinates (**Figure 4B**) (Kravitz et al., 2010). Analysis by fluorescence microscopy suggested a narrow range of nuclear labeling by Hoechst around the injection site (**Figure 4C**). The India Ink spot was used for visually guided microdissection of the DMS from acute brain slices. Hoechst 33258 allowed us to identify cells in the close vicinity of the precise injection coordinates by FACS (**Figure 4D**). Based on protocol optimization, 300 μm brain slices were prepared on the vibratome and digested with papain. Given the low cell density and high degree of interspersed white matter in the striatum, as well as the previously established density of MSN (Ena et al., 2013), we applied density gradient centrifugation to reduce the fraction of debris from the single-cell solution. Live cells were identified based on Hoechst 33258 stain and absence of propidium-iodide set against TdTomato, due to partial interference of their fluorescence spectra (**Figures 4D,E**). Finally, single D1 or D2 MSN were sorted into cooled 96-well PCR plates and immediately frozen to preserve the minute RNA amounts for scRNA-Seq.

### Single-Cell RNA-Seq From MSN of the Dorsomedial Striatum

We sought to molecularly characterize freshly isolated cells using our now standardized methods. To do so we decided to focus on RNA sequencing using a single cell approach. ScRNA-Seq methodologies are rapidly maturing especially in the area of specialized equipment to process the single cell suspensions. In our case, we decided to utilize well-plate based processing of each individual cell (Picelli et al., 2013). This approach has the drawback of increased cost and labor time associated with each sequenced cell but has the potential for lower bias as each cell that can be sorted by FACS into a well is able to be processed. Furthermore, the well-plate-based approach preserves potentially valuable flow cytometry data for each individual cell that can be used for cell characterization, such as fluorescent labels. We attempted to sequence 80 single cells and generated usable data from 77 cells (96%) with a median sequencing depth of 3.39 million reads per cell with 76.47% of reads aligning uniquely to the mouse genome (see Methods for details).

**FIGURE 5:**
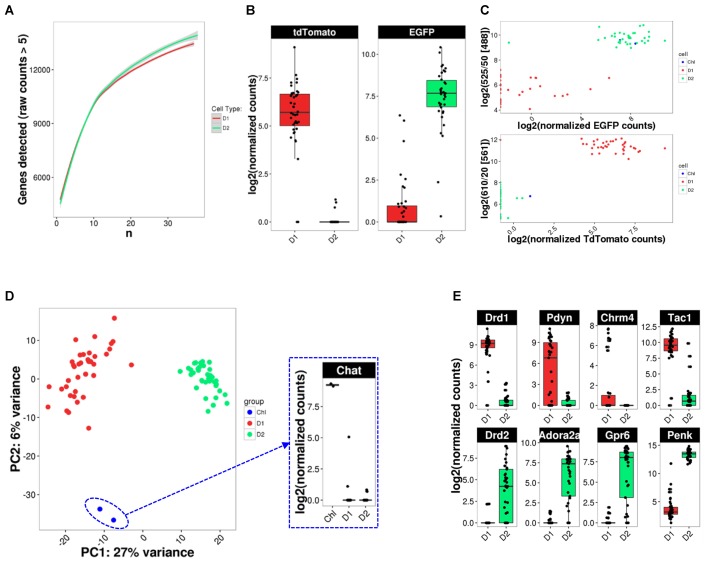
scRNA-Seq analysis identifies cell types. **(A)** Random down-sampling of cell numbers reveals relationship between stochasticity of gene expression in single cells and comprehensiveness of transcriptome capture with increasing cell numbers. Random sampling of defined cell numbers was repeated five times. **(B)** BAC-transgene expression in D1 and D2 MSN. **(C)** Comparison of reporter transgene fluorescence and mRNA levels. **(D)** PCA analysis for cell type identification reveals two *Drd2*-expressing cholinergic interneurons (ChI). Inset: *Chat* expression confirms cholinergic interneuron identity. **(E)** Box plots for previously reported MSN subtype-specific marker genes confirm subtype identity of analyzed MSN.

Given the previously described high variability and sparseness of single-cell gene expression data from adult CNS neurons (Zeisel et al., 2015), we first assessed whether our cell numbers for presumably homogeneous cell types allowed us to comprehensively measure the transcriptome (**Figure 5A**). Randomized sub-sampling of different numbers of cells and subsequent extrapolation suggests that with 40 single cells we capture greater than 95 percent of the transcriptome for both D1 and D2 MSN (**Figure 5A**). Examination of the fluorescent reporter gene expression for Drd2-EGFP and Drd1a-Tdtomato further suggested specificity to the respective MSN subtype (**Figure 5B**). This specificity was also reflected by the comparison of fluorescence as measured during FACS and fluorescent reporter gene counts as measured with scRNA-Seq (**Figure 5C**), confirming the validity and specificity of our cell labeling and isolation approach. Principal component analysis (PCA) using the top-100 most differentially expressed genes between the two MSN subtypes visually separated both MSN subtypes along their subtype identity (**Figure 5D**). In addition, PCA identified two Drd2-EGFP expressing cells as striatal cholinergic interneurons, based on their expression of choline-acetyltransferase (*Chat*). The appearance of Chat interneurons within the Drd2-EGFP expressing population was not surprising as the expression of the *Drd2* gene in this rare striatal interneuron type is well documented (Le Moine et al., 1990; Weiner et al., 1991). These two cells were excluded from subsequent analyses aimed at elucidating features of MSN subtypes. Also, PCA did not identify additional distinguishable subpopulations within the D1 and D2 MSN subtypes justifying their consideration as relatively homogeneous cell types for subsequent analyses. Finally, we examined differential expression of known MSN subtype markers that have been reported to distinguish D1 and D2 MSN (**Figure 5E**). Log2 fold expression (D1 over D2 MSN, with DESeq2-adjusted p) for *Drd1a* (9.71, *p* = 1.8E-131), *Pdyn* (9.47, *p* = 3.31E-28), *Tac1* (5.97, *p* = 9.64E-21), and *Chrm4* (8,42, *p* = 2.52E-6) in D1 MSN, and *Drd2* (-7.90, *p* = 3.83E-16), *Adora2a* (-9.76, *p* = 6.57E-39), *Penk1* (-6.41, *p* = 9.37E-36), and *Gpr6* (-11.03, *p* = 6.95E-38) in D2 MSN is largely consistent with previously established knowledge of the two MSN subtypes(Gerfen et al., 1990; Schiffmann and Vanderhaeghen, 1993; Hersch et al., 1995; Ince et al., 1997; Lobo et al., 2006).

### Comparison of scRNA-Seq Data to Population-Based MSN Reference Data

We next compared our scRNA-Seq data with previous, population-based reference microarray data for MSN subtypes. This allowed us to establish (I) how single-cell resolution data compares to population-based technologies and (II) whether single-cell data is able to reveal novel MSN subtype-specific genes that might be of interest for understanding MSN biology and potential therapeutic avenues in conditions involving MSN dysfunction. BacTRAP technology, which emerged from a combination of modified BAC-transgenes and translating ribosome affinity purification (TRAP), is currently the most cell type-specific, population-based gene expression technology (Doyle et al., 2008). Using BacTRAP mouse lines for *Drd1*-expressing (EGFPL10a, line CP73) and *Drd2*-expressing (EGFP-L10a, line CP101) CNS cell types, respectively, Heiman et al. (2008) have unearthed a wealth of previously unknown D1 and D2 MSN-specific genes and established a standard reference transcriptome for these two cell types.

**FIGURE 6:**
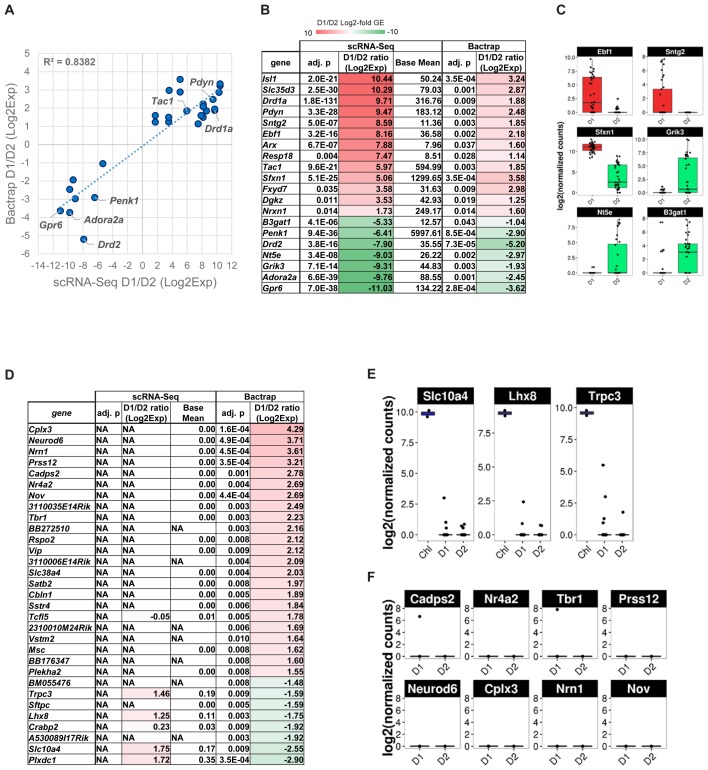
Gene expression specificity for MSN subtypes as measured by scRNA-Seq and compared to population-based gene expression data. BacTRAP data for this comparison was taken from Supplementary Table S2 by Heiman et al. (2008). **(A)** Scatter plot shows correlation for major MSN subtype-specific genes with adjusted *p* < 0.05 either for scRNA-Seq or BacTRAP. Notable examples are labeled. **(B)** Differential expression of major D1 and D2 MSN-specific genes commonly found by scRNA-Seq and reported by BacTRAP (adjusted *p* < 0.05). Genes displayed were found as statistically significant and with same specificity based on at least one microarray probe in the Heiman et al. (2008) data set. Genes are listed by differential expression from D1 MSN- (top, red) to D2 MSN-specific (bottom, green). **(C)** scRNA-Seq data of additional D1 and D2 MSN-specific markers identified by Heiman et al. (2008) using BacTRAP. **(D)** D1 and D2 MSN-specific gene expression patterns as identified by BacTRAP experiments that are not confirmed by scRNA-Seq. Genes with adjusted *p* < 0.01 based on BacTRAP are shown. For genes represented by several probes in the BacTRAP data, the adjusted *p*-value of the most significant probe is displayed. **(E)** Examples of reportedly D2 MSN-specific genes that are identified by scRNA-Seq as specific to striatal cholinergic interneurons. **(F)** Examples of reportedly D1 MSN-specific genes identified by scRNA-Seq as non-subtype-specific or not expressed in MSN.

We therefore compared differential gene expression lists from our scRNA-Seq experiment with the genes they reported as significantly differentially expressed between D1 and D2 MSN [**Figure 6A**, adjusted *p* < 0.05, Supplementary Table S2 of Heiman et al. (2008)]. Overall, we found a positive correlation for 20 genes that were reported significantly differentially expressed between D1 and D2 MSN by both data sets (*R*^2^ = 0.84). Although low in numbers, these overlapping genes between studies contain major known MSN subtype-specific markers including *Drd1a*, *Pdyn*, and *Tac1* for D1 MSN, and *Drd2*, *Adora2a*, *Penk1*, and *Gpr6* for D2 MSN (**Figures 6B,C**). However, the correlation was considerably lower when we compared all genes reported significant by BacTRAP, and also included by scRNA-Seq (*R*^2^ = 0.353, not shown). This was due in large part to a group of at least 83 genes that were not detected as significantly differentially expressed between D1 and D2 MSN by scRNA-Seq (**Figure 6D** and Supplementary Table S1). Furthermore, 49 of these 83 genes reported by Heiman et al. (2008) were not expressed at all in MSN according to our scRNA-Seq data. These discrepancies could suggest that by using scRNA-Seq we had missed a major group of MSN subtype-specific genes. We therefore analyzed these potentially false negatives in our scRNA-Seq data by comparing with an independent gene expression database, the Allen Brain Atlas (Lein et al., 2007). Based on this comparison, we noticed distinct patterns for reportedly D2 MSN-specific genes and D1 MSN-specific genes, respectively, that were not identified by scRNA-Seq. For the top-D2 MSN specific genes reported by BacTRAP technology and not identified by scRNA-Seq, *in situ* hybridization by the Allen Institute for Brain Science showed a sparse expression pattern in striatum that is not reminiscent of MSN (not shown), but rather of a rare striatal interneuron subtype, such as cholinergic interneurons. This was further corroborated by confirming their expression in the two cholinergic interneurons that were identified by PCA (**Figure 6E**, compare also to **Figure 5D**). These genes included *Lhx8*, which encodes a transcription factor important for striatal cholinergic interneuron development. For the 8 top-D1 MSN-specific genes reported by BacTRAP technology and not identified by scRNA-Seq, *in situ* hybridization by the Allen Institute for Brain Science suggests absence of expression in striatum, but high expression levels in cerebral cortex, particularly including layers L5 and L6 (**Figure 6F** and Supplementary Figure S4). These observations suggest that this group of 83 genes previously reported as MSN subtype-specific may not represent false negatives in our scRNA-Seq data, but are actually not differentially expressed between or not expressed at all in the D1 and D2 MSN subtypes.

**FIGURE 7:**
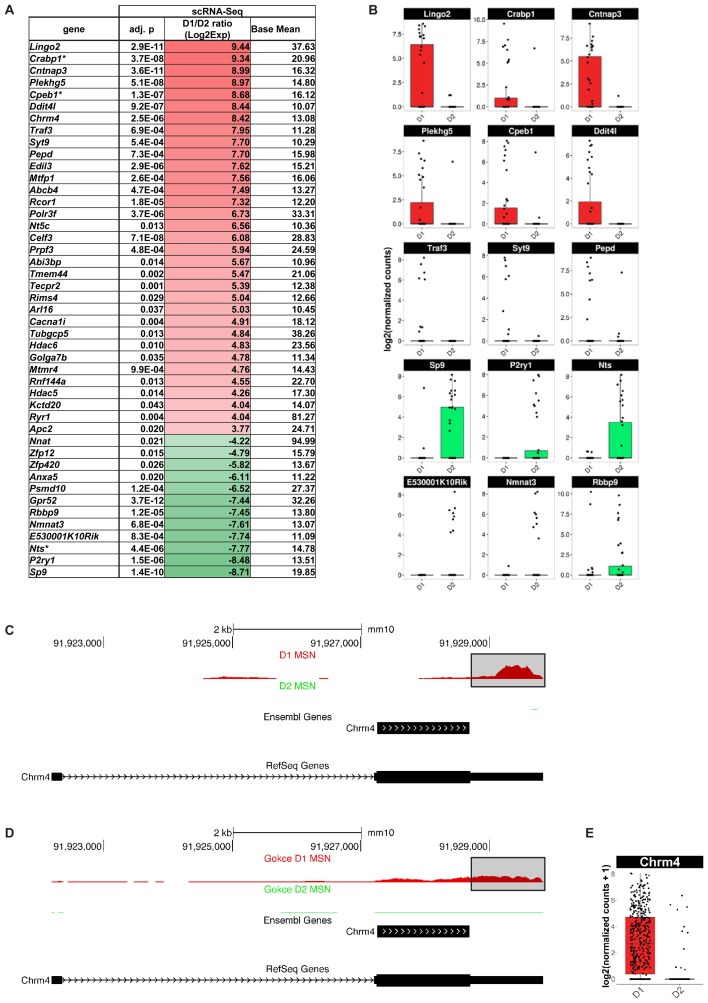
Medium spiny neurons (MSN) subtype-specific genes not found as differentially expressed by population-based gene expression studies. Shown are genes with a differential gene expression of >10-fold and a base mean of >10. **(A)** Genes listed by differential expression from D1 MSN- (top, red) to D2 MSN-specific (bottom, green). Asterisk (^∗^) indicates genes, which showed a tendency for the same MSN subtype in BacTRAP data but were not statistically significant in their differential gene expression (0.05 < *p* < 0.01). **(B)** Examples of differential gene expression between D1 and D2 MSN as assessed by scRNA-Seq. **(C)** *Chrm4* read alignments visualized and compared to two distinct gene set annotations, RefSeq and Ensembl. Reads from D1 (top) and D2 (bottom) MSN were separately aligned. No reads for Chrm4 were detected in D2 MSN. For D1 MSN, note the bias of read accumulation at the 3′ end of the *Chrm4* gene. The boxed area denotes gene expression signal only observed with the RefSeq gene set annotation, but not with Ensembl. **(D)** Read alignments in the *Chrm4* gene using scRNA-Seq data by Gokce et al. (2016). **(E)** Boxplot showing differential *Chrm4* gene expression between D1 and D2 MSN based on data from Gokce et al. (2016).

Finally, we explored whether scRNA-Seq actually identified any additional MSN subtype-specific genes compared to population-based gene expression analysis. We found 127 genes with significantly differential gene expression between D1 and D2 MSN of the dorsomedial striatum that, to our knowledge, have not been previously reported by any population-based transcriptome study in the adult mouse brain (**Figure 7**). Among the top D1 MSN-specific genes are *Lingo2* (*p* = 2.9E-11), *Cabp1* (*p* = 3.7E-8), *Cntnap3* (*p* = 3.6E-11), *Plekhg5* (*p* = 5.1E-8), *Cpeb1* (*p* = 1.3E-7) *Ddit4l* (*p* = 9.2E-07), and *Chrm4 (p* = 2.52E-6*)* (**Figures 7A,B**). Novel D2 MSN-specific genes include *Gpr52* (*p* = 3.7E-12), *Sp9* (*p* = 1.4E-10), *P2ry1* (*p* = 1.5E-06), and *Nts* (*p* = 4.4E-6) (**Figures 7A,B**). With exception of *Chrm4*, similar results were reported by a recent single-cell RNA-Seq study of striatal cell types (Gokce et al., 2016), thus emphasizing the importance of single-cell resolution for comprehensive assessment of cell type-specific genes.

The absence of the *Chrm4* gene in previous D1 MSN-specific gene expression data sets was surprising given multiple reports suggesting a D1-specific expression pattern of the M4 receptor among striatal MSN. This specificity was suggested at the behavioral and functional levels using knockout mice and pharmacology, and at the gene expression level using double *in situ* hybridization (Bernard et al., 1992; Ince et al., 1997; Jeon et al., 2010). We therefore quantified our scRNA-Seq data using two major gene set annotations used in the field, one curated by the NCBI RefSeq project (Refseq) and the one curated by the Ensembl genome database project (Ensembl; see also Methods), in order to examine the *Chrm4* locus in more detail (**Figure 7C**). Read pile-ups revealed the expected specificity of *Chrm4* expression to D1 as opposed to D2 MSN, consistent with our initial analysis. However, the majority of reads in our sequencing data aligned to the 3-prime untranslated region (3′-UTR) of *Chrm4*. This region is annotated in the RefSeq but not in the Ensembl gene set annotation. We repeated the same analysis on D1 and D2 MSN scRNA-Seq data from Gokce et al. (2016; **Figure 7D**), and the resulting read accumulation showed a slightly less pronounced, though still considerable 3′-end bias, and confirmed major expression of *Chrm4* in D1 MSN, but not in D2 MSN. Differential gene expression analysis using the Gokce data in combination with the more comprehensive RefSeq *Chrm4* gene annotation further corroborated significant *Chrm4* specificity to D1 MSN (*p* = 1.48E-86, **Figure 7E**). In summary, our scRNA-Seq experiments have revealed a set of novel MSN subtype-specific genes, including the D1 MSN-specific *Chrm4* gene, that are of interest for our understanding of the direct and indirect basal ganglia pathways.

## Discussion

The study of cell type-specific gene expression is of great interest for heterogeneous tissues such as the brain, both to increase our scientific understanding and the discovery of novel therapeutic targets. Functionally antagonistic MSN of the striatum have been at the center of this quest due to their relevance for major psychiatric, neurodegenerative and other CNS conditions. A major challenge in the endeavor gaining a better understanding of gene expression at single-cell resolution is the isolation of neurons from the heavily interconnected brain. Cell isolation is therefore a critical parameter that challenges comparison between studies as there are virtually no common standards for comparing cell viability and quality from complex tissue extracts. Here we established a robust and reproducible protocol for single cell isolation from adult rodent brain. We present novel approaches for cell labeling that, combined with state of the art stereotaxic surgery and FACS, pave the way toward reproducible cell type identification based on specific coordinates and independently of visible anatomical demarcations. Finally, we re-defined the MSN transcriptome using scRNA-Seq on D1 and D2 MSN and compared it to previously established standards based on bulk gene expression analyses. Our work revised previous data on MSN subtype specific gene expression and added to the list of MSN subtype-specific marker genes, which together emphasized the need for single-cell resolution gene expression studies to fully resolve the cellular complexity of the brain.

Our study presents an in-depth assessment of common steps and factors potentially affecting the efficacy and quality of single cell isolation from adult rodent brain for downstream assays including RNA-Seq. Combinations of cell-permeant and non-cell-permeant nuclear dyes are useful to accurately measure the fraction of dead cells in single-cell suspensions from adult brain that are inherently heterogeneous due to abundant debris particles from neuropil and myelin. Labeling of cells with cell permeant nuclear dyes including DRAQ5 or Hoechst 33258 improves separation of cell bodies from debris by FACS to an extent superior to classic forward and side scatter analysis (FSC/SSC). In addition, this methodology enables precise assessment of cell viability in combination with established live/dead stains, independently of the degree of debris contributing to total particle numbers as detected by flow cytometry. Using these improved quantitative methods for cell analysis, we identified major sources of variability in cellular recovery around initial steps of vibratome sectioning and subsequent enzymatic digestion of brain tissue. Surprisingly, the thickness of tissue slices played a major role in the recovery of intact cells, and more interestingly even, thinner brain slices yielded more intact cells compared to thicker ones. This may be counter-intuitive as thicker slices have a smaller surface/volume ratio and thus may expose fewer cells to disruptive forces of the vibratome blade. This result could therefore indicate that other parameters play a role, such as enzyme accessibility to soften cellular connectivity deeper within the tissue, or access to nutrients and gas exchange from the surrounding medium during the procedure that help prevent cell death in thinner tissue slices. In turn, parameters of trituration (inner diameter of needles, differences of manual trituration by different individuals) showed no significant effect on overall performance, thus corroborating the robustness of the protocol. One possible interpretation of this outcome is that with advanced trituration steps the increasing shear forces by smaller needle diameters are counterbalanced by decreasing size of remaining tissue clumps. In such a scenario, cells exposed at the surface of remaining tissue pieces would be exposed to comparable forces across the trituration protocol. More work to understand the mechanisms acting during the transition from interconnected tissue to single cell suspension will be needed. In conclusion, our proposed protocol facilitates the measurement of critical parameters for assessments of single-cell suspensions and thus contributes to comparability of results from downstream assays including scRNA-Seq.

Applying our protocol to striatal MSN, we develop a method compatible with state-of-the-art stereotaxic surgery to label and isolate cells from a functionally distinct sub-region of the striatum, the DMS. Comparison of scRNA-Seq data from D1 MSN and D2 MSN confirm several previous findings about these cell types and their isolation using BAC-transgenic reporter technology. First, the discovery of two cholinergic interneurons among the *Drd2-EGFP* labeled cells confirms the specificity of this BAC-transgenic EGFP reporter line for all *Drd2* expressing striatal cell types beyond MSN. The transcriptional signature of striatal cholinergic interneurons is therefore expected to be present in previously reported D2 MSN-specific transcriptomes from bulk striatal tissue in combination with transgenic lines that are based on the same or highly similar BAC clones around the *Drd2* gene locus. As we showed by comparing to data from BacTRAP studies this likely resulted in previous confusion of expression pattern for cholinergic-interneuron-specific genes including *Lhx8*, *Slc10a4*, or *Trpc3*. Second, our data resolve inconsistencies arising from a list of presumably D1 MSN-specific marker genes reported by the BacTRAP studies, which by other reports were found highly specific to projection neurons of the deeper cortical layers, including *Cplx3*, *Tbr1*, and *Neurod6*. Interestingly, *Drd1a* expression is wide-spread across excitatory projection neurons of the deeper cortical layers, a fact that also applies to the *Drd1a-TdTomato* BAC transgenic reporter used in our study (see also **Figure 4B**). Therefore, a possible explanation for appearance of cortical excitatory markers in a presumably D1 MSN-specific transcriptome, as assessed by BacTRAP technology, is a “contamination” by ribosome-bound mRNAs from abundant cortical excitatory axons that project to the striatum, and through the striatum to other CNS regions and beyond. Finally, we cannot exclude the possibility that our MSN transcriptome originating specifically from the DMS might slightly differ from MSN transcriptomes of other striatal sub-regions. However, good correlation of our MSN data with the recent scRNA-Seq study by Gokce et al. (2016) suggests a minor impact of regional specificity on the differences observed between our results and previous MSN transcriptomes that originated from population-based approaches, such as by BacTRAP technology. Our study therefore emphasizes the value of single-cell technologies in gaining profound understanding of the brain’s cellular complexity and for the resolution of potential short-comings due to challenges of specific cell labeling and isolation techniques.

In addition to resolving previous inconsistencies between the MSN transcriptomes from different studies, we present a list of MSN subtype-specific marker genes that have not been observed with bulk-tissue transcriptomic approaches. Some of these have been described in the recent single-cell study of striatal cell types by Gokce et al. (2016) including the *Sp9* gene. *Chrm4* has previously been suggested to be D1 MSN-specific by studies using other techniques, but to our knowledge, has not been mentioned in both landmark mRNA expression studies in MSN mentioned above (Bernard et al., 1992; Ince et al., 1997). Using a different, more comprehensive gene set annotation for quantification, our analyses firmly place *Chrm4* within D1 MSN-specific genes. We further provide evidence that the use of different gene set annotations in combination with a 3′-end sequencing bias was a likely cause for *Chrm4* escaping previous attempts at defining the MSN subtype-specific transcriptome by scRNA-Seq. The Ensembl gene set annotation lacks *Chrm4* UTRs, including a non-coding upstream exon. In contrast, the RefSeq gene set annotation includes *Chrm4* UTRs. Our data as well as the data by Gokce et al. showed a general bias in signal strength for the 3′-end of the transcript including the differentially annotated 3′-UTR. This finding is not unexpected given that many low-input cDNA generation technologies (including SmartSeq V3 used in this study and V2 used by Gokce et al., 2016) result in a 3′-end bias particularly for long transcripts (Picelli, 2016). However, the finding may point at a general challenge that gene expression quantification by scRNA-Seq faces in light of yet incomplete gene set annotation.

Together, our observations resolve apparently inconsistent findings regarding the *Chrm4* gene expression specificity. More importantly, they align the D1 MSN-specific action of its gene product, the M4 muscarinic acetylcholine receptor, with previous studies of M4 receptor action in the basal ganglia. Activation of the G_αi_-coupled M4 receptor is expected to have an attenuating effect on neuronal output. Several knockout and pharmacological studies have suggested a negative effect of M4 receptor action on striatal dopamine activity (Gomeza et al., 1999; de la Cour et al., 2015). Given the re-enforcing (positive) response of the direct basal ganglia pathway resulting from D1 MSN responding to dopamine, this M4-mediated attenuation would make sense in light of M4 receptor expression specifically in D1 MSN. Additional evidence for this comes from a study showing that conditional *Chrm4* knockout specifically in D1-MSN recapitulates the major forebrain-related phenotypes of the full *Chrm4* knockout (Gomeza et al., 1999; Jeon et al., 2010). Finally, given the proposed importance of striatal cholinergic action through the M4 receptor for modulation of cortico-striatal plasticity and dopamine action in the context of schizophrenia (Watt et al., 2013), Parkinson’s disease (Shen et al., 2016), Huntington’s disease (Pancani et al., 2015), and substance abuse (de la Cour et al., 2015), the example of *Chrm4* illustrates the potential of scRNA-Seq for the discovery of cell type-specific drug targets in the CNS.

## Author Contributions

TP, MH, SB, and KT conceived the study. HH, MW, AN, EZ, HL, DL, and TP performed the cell collection and flow cytometry experiments. AS conducted the scRNA-Seq experiments. MDB, SS, JN, DL, and TP analyzed the data. DZ and HH performed the stereotaxic surgeries. TP designed the experiments and wrote the manuscript.

## Conflict of Interest Statement

TP, SB, KT, HH, MW, AN, EZ, HL, DL, SS, JN, and DZ are or were full-time employees at Circuit Therapeutics, Inc. when this study was conducted. The remaining authors declare that the research was conducted in the absence of any commercial or financial relationships that could be construed as a potential conflict of interest.
