# Supplementary material for: A Guide to Single-Cell Transcriptomics in Adult Rodent Brain: The Medium Spiny Neuron Transcriptome Revisited
Source: Front Cell Neurosci. 2018 Jun 15;12:159. doi: 10.3389/fncel.2018.00159 (PMC6018757; doi:10.3389/fncel.2018.00159)
Supplement: Supplementary file 1 [file Presentation_1.pdf]

Supplementary Material

A Guide To Single-Cell Transcriptomics In Adult Rodent Brain: The Medium Spiny Neuron Transcriptome Revisited

Authors:
Hanson Ho[1,§], Matt De Both[2,§], Ashley Siniard[2], Sasha Sharma[1], James H Notwell[1], Michelle Wallace[1], Dino P. Leone[1,3], Amy Nguyen[1], Eric Zhao[1], Hannah Lee[1], Daniel Zwilling[1], Kimberly R. Thompson[1], Steven P Braithwaite[3], Matthew Huentelman[2], and Thomas Portmann*[1]

Affiliations:
[1] Circuit Therapeutics Inc, Menlo Park, CA, USA
[2] Translational Genomics Research Institute (TGen), Neurogenomics Division, Phoenix, AZ, USA
[3] current: Alkahest Inc, San Carlos, CA, USA
[§] equal contributors
*correspondence: tportmann@circuittx.com

Supplementary Figures and Tables

Figure S1. Assessment of cell morphology and viability after tissue dissociation. Cells were exposed to enzymatic digestion according to two published protocols by Brewer et al. (Papain, top) and Ena et al. (Protease Type XIII, bottom). Phase contrast (Phase) and fluorescence (PI) images for 8 representative cells are shown. PI: propidium-iodide

Figure S1

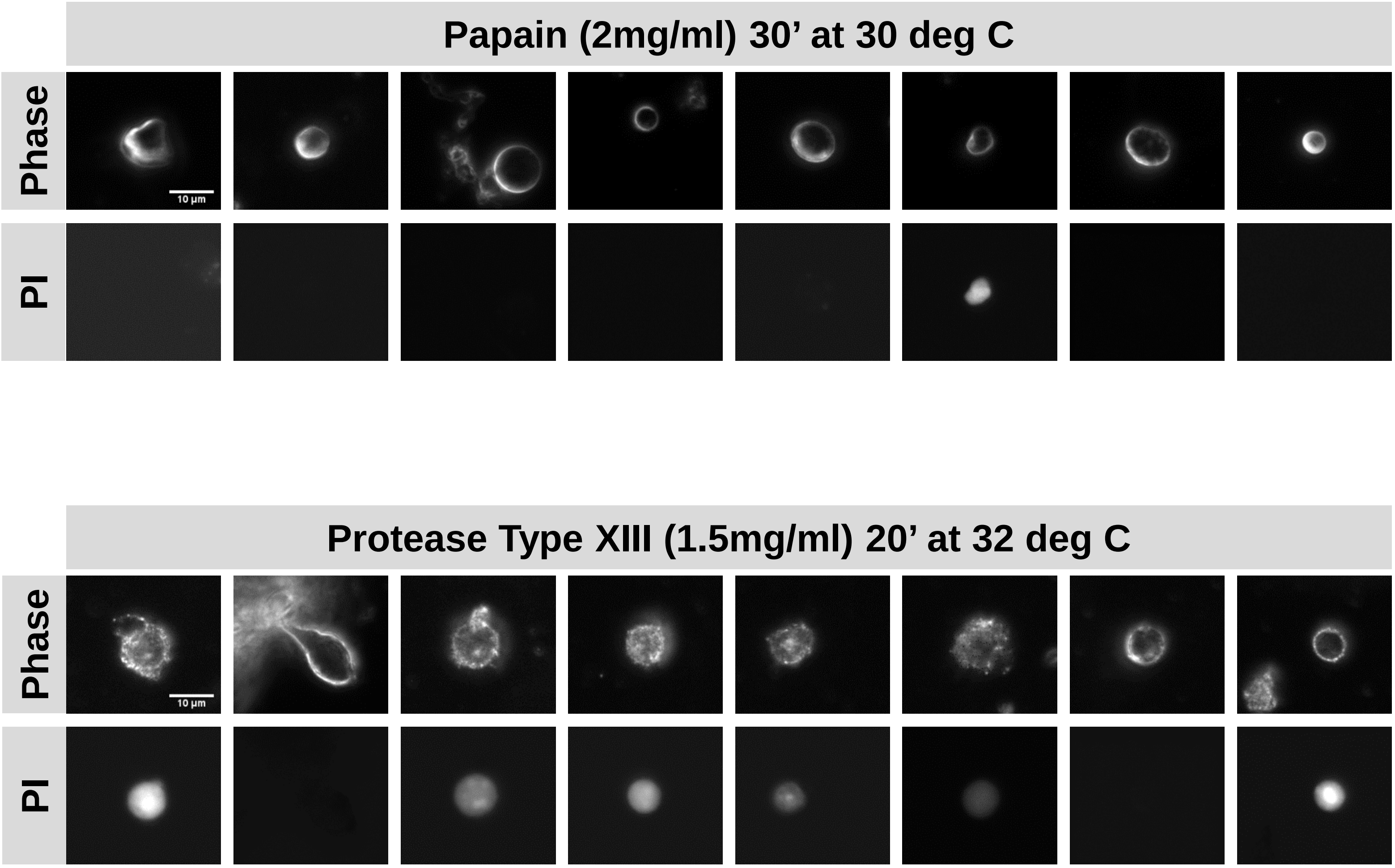
Papain (2mg/ml) 30’ at 30 deg C
Phase
PI
10 µm
Protease Type XIII (1.5mg/ml) 20’ at 32 deg C
Phase
PI
10 µm

Figure S2. Trituration and density gradient centrifugation. A) Schematic view of experimental design to assess impact of trituration steps with decreasing needle inner diameter (ID). Key variables included thickness of tissue slices, and number of trituration steps. Two independent experiments were performed. B-C) Impact of tissue slice thickness, trituration steps (trit.) and experiment (Exp) on yield of singlets (B), and live single cells (C). Nested ANOVA was performed with individuals and experiments treated as replicate groups. D) Phase contrast images of particle contents from the density gradient (left) and supernatant (right). Note the presence of smaller cells in the debris layer from supernatant (arrow heads). E) Representative flow cytometry data (n=8000 forebrain cells) from the comparison of enzyme treatment for tissue dissociation. Tissue treatment with papain yields greater numbers of intact (live) cells than treatment with pronase.

Figure S2

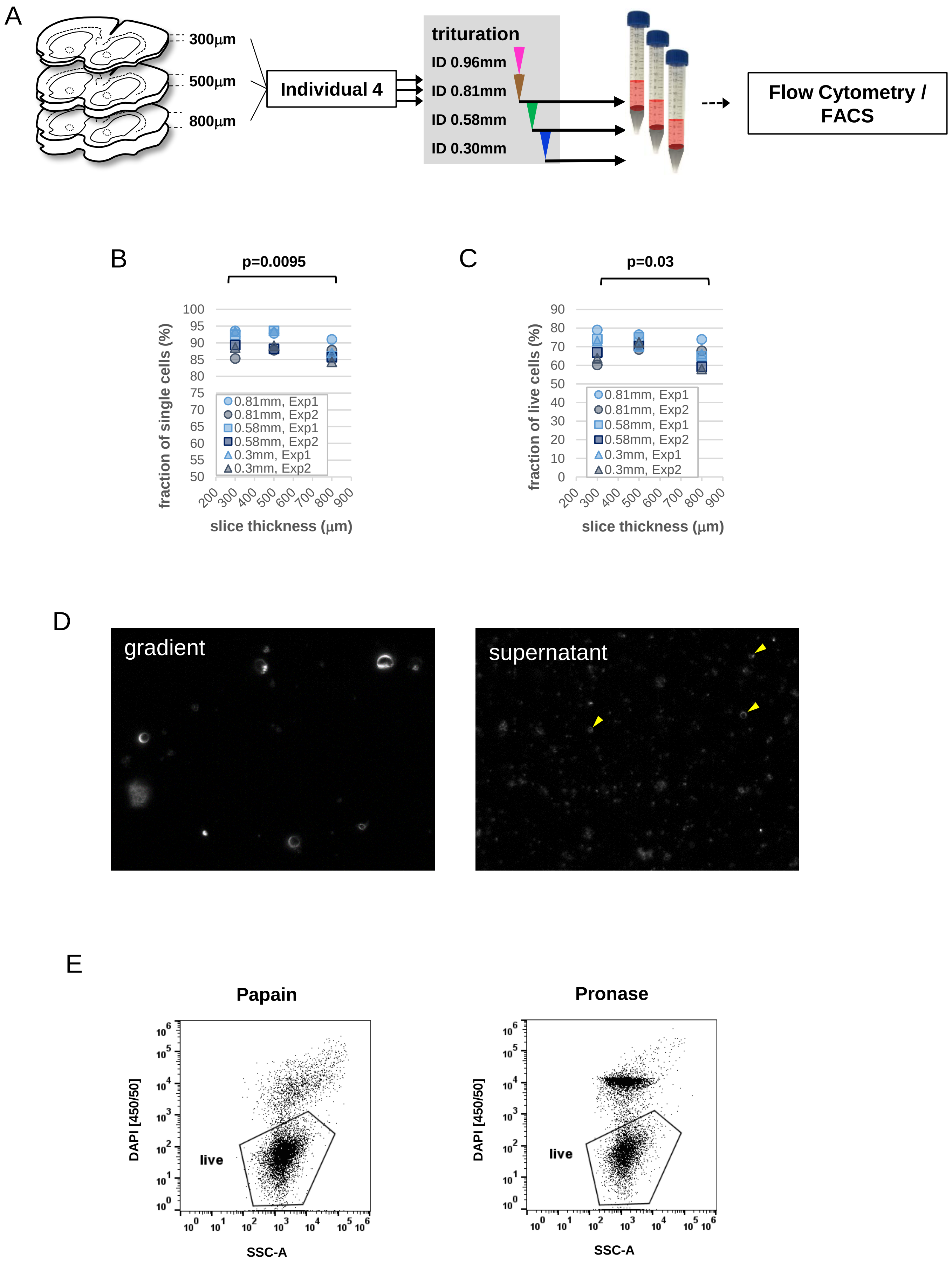
A
300µm
500µm
800µm
Individual 4
trituration
ID 0.96mm
ID 0.81mm
ID 0.58mm
ID 0.30mm
Flow Cytometry / FACS
B
p=0.0095
fraction of single cells (%)
slice thickness (µm)
0.81mm, Exp1
0.81mm, Exp2
0.58mm, Exp1
0.58mm, Exp2
0.3mm, Exp1
0.3mm, Exp2
C
p=0.03
fraction of live cells (%)
slice thickness (µm)
D
gradient
supernatant
E
Papain
Pronase
DAPI [450/50]
SSC-A
live

Figure S3. Visualization of fluorescent labels. Images of fluorescently labelled MSN isolated by FACS. Note the round shape of cells and in some cases, remnants of neurites attached to them. Labels are as follows, DRAQ5 (blue): cell nuclei, Drd1a-TdTomato (red): D1 MSN, Drd2-EGFP (green): D2 MSN.

Figure S3

A

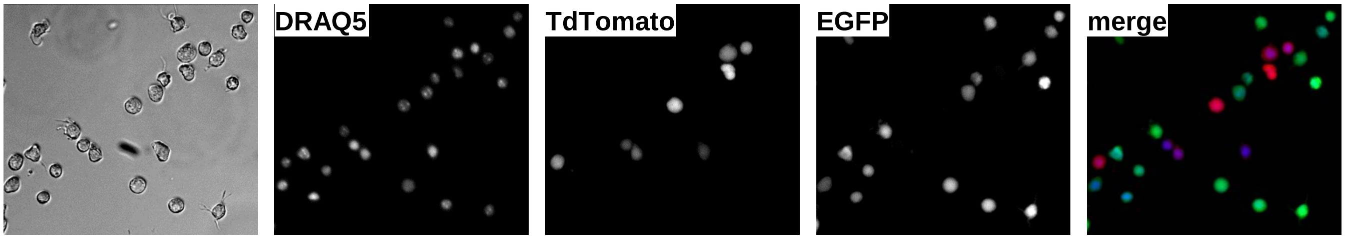

Figure S4. Confirmation of cortical GE for previously reported D1 MSN-specific genes. *In situ* hybridization experiments showing examples of genes that are expressed in cortex, especially deeper cortical layers, while largely absent in striatum. Image source: Allen Brain Atlas (adult mouse brain) by Allen Institute for Brain Science. cc: corpus callosum, ms: medial septum. Arrowheads indicate regions of particularly high expression around cortical layers L5 and/or L6.

Figure S4

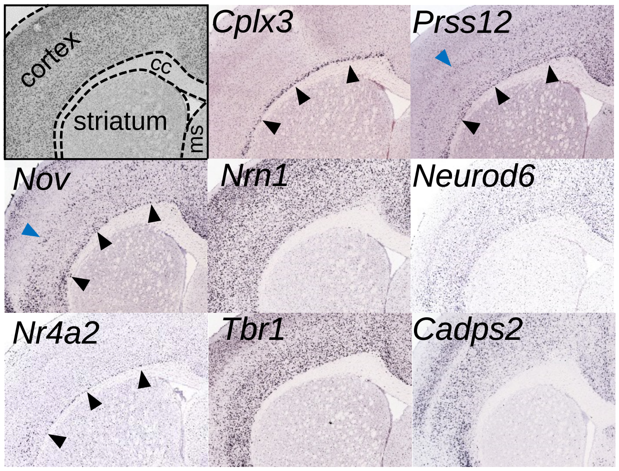

Allen Institute for Brain Science

Table S1. Genes not differentially expressed between MSN subtypes based on scRNA-Seq. List of genes that were previously reported as differentially expressed between D1 and D2 MSN by Heiman et al. ScRNA-Seq data suggests they are not differentially expressed or not expressed at all in D1 and D2 MSN.

Table S1

Affymetrix Probe set ID	GenBank	Gene Symbol	Ensembl ID	alternative name
1456146_at	BI735554	2210411A11Rik		
1428184_at	BB348639	3110035E14Rik	ENSMUSG00000067879	
1438112_at	AA546727	9430021M05Rik	ENSMUSG00000054457	
1457032_at	BB546359	Ak5	ENSMUSG00000039058	
1435959_at	BM246535	Arhgap15	ENSMUSG00000049744	
1437091_at	AV323885	Asic4	ENSMUSG00000033007	Accn4
1436503_at	BF302511	BC048546	ENSMUSG00000047228	
1451620_at	BB768838	C1ql3	ENSMUSG00000049630	
1451499_at	AF000969	Cadps2	ENSMUSG00000017978	
1417605_s_at	NM_133926	Camk1	ENSMUSG00000030272	
1423287_at	AA016422	Cbln1	ENSMUSG00000031654	
1454770_at	AV221910	Cckbr	ENSMUSG00000030898	
1451322_at	BC024580	Cmbl	ENSMUSG00000022235	
1419517_at	NM_028408	Cnih3	ENSMUSG00000026514	
1424606_at	BC024854	Cplx3	ENSMUSG00000039714	
1451191_at	BC018397	Crabp2	ENSMUSG00000004885	
1428283_at	AK004699	Cyp2s1	ENSMUSG00000040703	
1454659_at	BG069699	Dctd	ENSMUSG00000031562	
1448669_at	AK004853	Dkk3	ENSMUSG00000030772	
1436862_at	BB543070	Doc2a	ENSMUSG00000052301	
1434695_at	AV270035	Dtl	ENSMUSG00000037474	
1422586_at	NM_021306	Ecel1	ENSMUSG00000026247	
1453558_at	AK015697	Efcab10	ENSMUSG00000020562	
1431339_a_at	AK007560	Efhd2	ENSMUSG00000040659	
1443827_x_at	BB375974	Fam20c	ENSMUSG00000025854	
1419577_at	NM_133999	Fig4	ENSMUSG00000038417	A530089I17Rik
1417343_at	AB032010	Fxyd6	ENSMUSG00000066705	
1440361_at	BB272510	Gm12371	ENSMUSG00000084898	A830076I15Rik
1455885_at	AV238106	Gna12	ENSMUSG00000000149	
1447669_s_at	AV347903	Gng4	ENSMUSG00000021303	
1428323_at	BQ175968	Gpd2	ENSMUSG00000026827	
1443129_at	BB363699	Gpr139	ENSMUSG00000066197	
1452540_a_at	M25487	Hist1h2bp	ENSMUSG00000069308	
1439614_at	BB308379	Htr4	ENSMUSG00000026322	
1448839_at	NM_030697	Kank3	ENSMUSG00000042099	Ankrd47
1425090_s_at	BC024837	Kcnc4	ENSMUSG00000027895	
1427300_at	D49658	Lhx8	ENSMUSG00000096225	
1429274_at	AK009282	Lypd6b	ENSMUSG00000026765	2310010M24Rik
1421926_at	AV329330	Mapk11	ENSMUSG00000053137	
1441388_at	BB428710	Mbd2	ENSMUSG00000024513	
1457277_at	AI314927	Mblac1	ENSMUSG00000049285	
1426937_at	AK018128	Medag	ENSMUSG00000029659	
1418417_at	NM_010827	Msc	ENSMUSG00000025930	
1427115_at	M74753	Myh3	ENSMUSG00000020908	
1437156_at	BB392041	Necab1	ENSMUSG00000040536	Efcbp1
1418047_at	NM_009717	Neurod6	ENSMUSG00000037984	
1426852_x_at	X96585	Nov	ENSMUSG00000037362	
1447863_s_at	BB322941	Nr4a2	ENSMUSG00000026826	
1428393_at	AK003046	Nrn1	ENSMUSG00000039114	
1425784_a_at	D78264	Olfm1	ENSMUSG00000026833	
1417288_at	NM_031257	Plekha2	ENSMUSG00000031557	
1424902_at	AF378760	Plxdc1	ENSMUSG00000017417	
1422673_at	NM_008858	Prkd1	ENSMUSG00000002688	Prkcm
1429269_at	BE992549	Prr36	ENSMUSG00000064125	BC068157
1420388_at	NM_008939	Prss12	ENSMUSG00000027978	
1417400_at	NM_030690	Rai14	ENSMUSG00000022246	
1421144_at	NM_023879	Rpgrip1	ENSMUSG00000057132	
1424763_at	BC019423	Rsph9	ENSMUSG00000023966	Rsph9
1455893_at	BG067392	Rspo2	ENSMUSG00000051920	
1439573_at	BE992565	Rtn4rl2	ENSMUSG00000050896	
1427017_at	BB104560	Satb2	ENSMUSG00000038331	
1448415_a_at	NM_009153	Sema3b	ENSMUSG00000057969	
1418639_at	NM_011359	Sftpc	ENSMUSG00000022097	
1433578_at	BE824538	Slc10a4	ENSMUSG00000029219	
1448889_at	NM_027052	Slc38a4	ENSMUSG00000022464	
1437231_at	AV246497	Slitrk6	ENSMUSG00000045871	
1438729_at	BB331017	Sox1	ENSMUSG00000096014	BB176347
1451342_at	BC020531	Spon1	ENSMUSG00000038156	
1415849_s_at	BC010581	Stmn1	ENSMUSG00000028832	
1416711_at	NM_009322	Tbr1	ENSMUSG00000035033	
1456515_s_at	AV044715	Tcfl5	ENSMUSG00000038932	
1429175_at	AK014196	Tmem178	ENSMUSG00000024245	
1441917_s_at	BB468188	Tmem40	ENSMUSG00000059900	
1417192_at	NM_138599	Tomm70a	ENSMUSG00000022752	
1455739_at	BB279146	Tpbgl	ENSMUSG00000096606	Gm4980
1449577_x_at	AK003186	Tpm2	ENSMUSG00000028464	
1419879_s_at	AA960166	Trim25	ENSMUSG00000000275	
1417577_at	NM_019510	Trpc3	ENSMUSG00000027716	
1452779_at	AK014009	Ube2ql1	ENSMUSG00000052981	3110006E14Rik
1428664_at	AK018599	Vip	ENSMUSG00000019772	
1452065_at	BB085570	Vstm2a	ENSMUSG00000048834	
1449314_at	NM_011766	Zfpm2	ENSMUSG00000022306	
1423477_at	BB361162	Zic1	ENSMUSG00000032368	
